# Use of rifampin in persistent coagulase negative staphylococcal bacteremia in neonates

**DOI:** 10.1186/1471-2431-10-84

**Published:** 2010-11-19

**Authors:** N Margreth van der Lugt, Sylke J Steggerda, Frans J Walther

**Affiliations:** 1 Division of Neonatology, Department of Pediatrics, Leiden University Medical Center, Leiden, The Netherlands

## Abstract

**Background:**

Coagulase negative staphylococci (CoNS) are the most common cause of neonatal sepsis in the Neonatal Intensive Care Unit (NICU). A minority of neonates does not respond to vancomycin therapy and develops persistent bacteremia, which may be treated with rifampin. We evaluated the use of rifampin in persistent CoNS bacteremia.

**Methods:**

Retrospective study of 137 neonates with CoNS bacteremia during admission to a tertiary NICU between July 2006 and July 2009. Main outcome measures were total duration of bacteremia and the adequacy of vancomycin and rifampin therapy.

**Results:**

137/1696 (8.0%) neonates developed a CoNS bacteremia. Eighteen were treated with rifampin because of persistent bacteremia (3 positive blood cultures at least 48 hours apart with clinical symptoms) or (a serious suspicion of) an intravascular thrombus. Duration of bacteremia prior to rifampin therapy (8.0 ± 3.6 days) was positively correlated (p < 0.001) to the total duration of bacteremia (10.3 ± 3.7 days). After starting rifampin therapy C-reactive protein (CRP) levels of all neonates declined and blood cultures became sterile after 2.3 ± 1.6 days. Vancomycin levels were not consistently measured in all neonates, resulting in late detection of subtherapeutic trough levels.

**Conclusion:**

Rifampin may be effective in the treatment of persistent CoNS infections in neonates. Outcome may be improved by adequate monitoring of vancomycin trough levels.

## Background

Sepsis due to coagulase negative staphylococci (CoNS) is common in the neonatal intensive care unit (NICU). The incidence of CoNS sepsis varies between 1.3 and 19.9%, depending on birth weight and gestational age [1-5]. Most of these infections respond well to vancomycin, the first drug of choice. A minority of neonates develops a persistent staphylococcal bacteremia, which does not respond to vancomycin. For these neonates rifampin may be a safe and effective additive treatment to vancomycin [1,6-8]. Interaction between vancomycin and rifampin in treatment of staphylococcal infections is ambiguous, as some studies demonstrate antagonism and others synergism or indifference [9-12]. High concentrations of rifampin may result in antagonism [13,14]. Rifampin is only effective as combination therapy, because resistance develops when rifampin is used as monotherapy [15].

Through its highly lipophilic character, rifampin molecules can easily cross biological membranes, resulting in a wide tissue distribution [6,16]. The efficacy of rifampin in persistent staphylococcal bacteremia is due to its abilities to enhance serum bactericidal activity and to penetrate phagocytic leukocytes for intraleukocytic killing of staphylococci [17].

Pharmacokinetic research has demonstrated a positive correlation between the duration of rifampin therapy and its clearance, the equilibrium clearance is achieved after one to two weeks. The increase in clearance and decrease in half-life are probably due to auto-induction of the metabolism of rifampin and require caution to maintain serum levels within the therapeutic range by adjusting the dose of rifampin, when necessary [6,16,18].

Although CoNS bacteremia is common in NICUs and the treatment of persistent CoNS bacteremia with rifampin seems successful, previous studies were only small case reports or studies focusing on pharmacokinetics. The aim of this study was to evaluate the existing local guidelines for the use of rifampin therapy in persistent CoNS infection, checking the current indications to start rifampin therapy and estimating its efficacy.

## Methods

### Study population

The study population of this retrospective chart review consisted of all neonates admitted to the neonatology department of the Leiden University Medical Center (LUMC) between July 2006 and July 2009. The Medical Ethics Committee of the LUMC did not require approval of this study because it consisted of retrospective chart review, nor did the medical ethics committee require written consent by the parents for their infant's information to be stored in the hospital database and used for research. Approval by the ethics committee and informed consent was not necessary as the patient data were analyzed anonymously.

Inclusion criterion was the presence of a positive blood culture for CoNS. Persistent CoNS bacteremia was defined as 3 positive blood cultures, spaced at least 48 hours apart, in combination with clinical symptoms of sepsis. The indication to start rifampin treatment was persistent CoNS bacteremia despite treatment with vancomycin and removal of indwelling catheters, or a non-persistent CoNS bacteremia in combination with a proven intravascular thrombus. Starting dose of rifampin was 10 mg/kg/day intravenously.

### Data collection

Data on demographic, perinatal and postnatal clinical characteristics were collected to provide an overview of baseline characteristics and included birth weight, gestational age, gender, exposure to prenatal and postnatal steroids, presence of chorioamnionitis, hyperglycemia, prolonged rupture of membranes (PROM), asphyxia, respiratory distress syndrome (RDS)[19], bronchopulmonary dysplasia (BPD)[20], necrotizing enterocolitis (NEC)[21], cystic periventricular leukomalacia (PVL)[22] and intraventricular hemorrhage (IVH)[23,24]. These data were collected from the neonatal charts and used to compare neonates with non-persistent and persistent CoNS bacteremia.

Primary outcome measures were the total duration of bacteremia and the adequacy of vancomycin treatment, estimated by following trough levels obtained after the initiation of vancomycin therapy until the tenth day of rifampin therapy. The desired range of trough levels of vancomycin was 5-10 mg/L, trough levels <5 mg/L were considered to be subtherapeutic and the desired range for peak levels was 20-30 mg/L. Other variables studied included plasma urea and creatinine levels and duration of vancomycin therapy.

Main outcomes for analysis of the group of rifampin treated neonates were total duration of bacteremia and rapidity of sterilization of blood cultures after the start of rifampin. Age at start of infection, CRP levels from the first day of CoNS positive blood culture until the tenth day of rifampin treatment, and duration and dose of rifampin treatment were additional variables among rifampin treated neonates.

Identification of CoNS isolates was performed by the microbiology department using Bactec Peds Plus bottles (Becton and Dickinson, Franklin Lakes, NJ USA). Blood cultures, complete blood count and CRP were drawn upon clinical suspicion of sepsis. CRP levels were determined daily during therapy with antibiotics. Vancomycin serum samples were drawn just before the third dose and 1 hour after administration of the third dose. When the dosage of vancomycin was changed, another serum sample was drawn around the second dose after the change. CRP levels were measured using a immunoturbidimetric assay (imCRP, detection limit ≥3 mg/L [25]) and serum vancomycin levels by a fluorescence polarization assay [26].

CoNS bacteremia was an indication for removal of central venous lines and sonography for a remaining vascular thrombus.

### Statistical analyses

Data are reported as mean values ± standard deviation, minimum and maximum, numerical values or categories. Analyses were performed with SPSS Version 16.0 (SPSS Inc., Chicago, IL). Numerical data were analyzed by bivariate Pearson correlation and unpaired T-tests, categorical data were analyzed using a chi-squared test. To correct for potential confounding effects, logistic regression analysis was done.

## Results

In the period between July 2006 and July 2009 1696 neonates were admitted to the NICU with a mean birth weight of 1271 ± 663 gram and a gestational age of 29.2 ± 3.2 weeks. The incidence of CoNS bacteremia was 137/1696 (8%), 17 (12%) of these neonates developed a persistent CoNS bacteremia and in 3 of them an intravascular thrombus was identified. One neonate with a CoNS sepsis also had a S. aureus sepsis.

**Figure 1:**
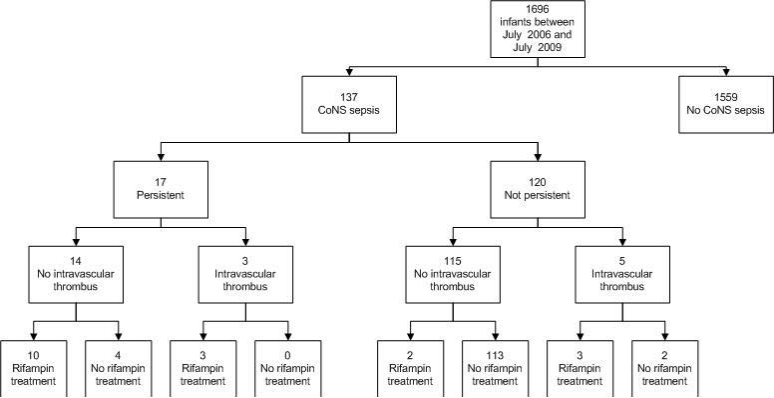
**Flow chart of included patients**.

A flowchart of the included patients can be seen in figure 1. Baseline characteristics of the included patients are listed in table 1. Newborn infants with persistent CoNS bacteremia had lower birth weights (p = 0.008) and, independent of birth weight, more often hyperglycemia (p = 0.007), than infants with non-persistent CoNS bacteremia. Subtherapeutic vancomycin trough levels were equally divided among the groups with persistent and non-persistent CoNS bacteremia (p = 0.712).

**Table 1:** Baseline characteristics of all included patients.

	Non-persistent CoNS bacteremia N = 120	Persistent CoNS bacteremia N = 17	P-value
Prenatal Steroids			0.668
0 doses	50 (43.1%)	9(52.9%)	
1 dose	47 (40.5%)	5 (29.4%)	
2 doses	19 (16.4%)	3 (17.6%)	

Chorioamnionitis*	9 (7.8%)	1 (5.9%)	0.784

PROM**	14 (12.0%)	2 (11.8%)	0.981

Asphyxia***	7 (5.8%)	0 (0%)	0.307

Gestational age, weeks	29.4 ± 3.3	28.0 ± 2.3	0.093

Birth weight, g	1,327 ± 686	874 ± 204	*0.008*

Gender (male)	71 (59.2%)	10 (58.8%)	0.979

			**Multivariate** **P-value** **(corrected for birth weight)**
Hyperglycemia****	8 (6.7%)	8 (47.1%)	*0.007*

IVH grade 3/4	9 (7.5%)	0 (0.0%)	0.999

Cystic PVL	2 (1.7%)	1 (5.9%)	0.640

NEC grade 2/3	4 (3.3%)	2 (11.8%)	0.766

RDS grade 3/4	23 (19.2%)	7 (41.2%)	0.788

BPD*****	26 (21.7%)	9 (52.9%)	0.771

Postnatal steroids	7 (5.8%)	4 (23.5%)	0.652

Died during admission	3 (2.5%)	1 (5.9%)	0.754

* Smelly amniotic fluid, maternal fever or signs of infection at birth

** Rupture of membranes >24 hours

*** Presence of minimal 3 criteria:

1) Decelerative CTG or meconium containing amniotic fluid

2) Umbilical cord pH <7.10

3) Apgar score <5 after 5 minutes

4) Spontaneous respiratory depression >5 minutes after birth

5) Multiple organ failure

**** Glucose levels of >10 mmol/L during >12 hours, treated with insulin >12 hours

***** Need for oxygen-therapy at a gestational age of 36 weeks or at discharge

Eighteen of the 137 neonates received rifampin treatment, started after 8.0 ± 3.6 (mean ± SD) days of CoNS bacteremia. 13/18 neonates had persistent CoNS bacteremia (in three of them an intravascular thrombus was found), 3/18 had an intravascular thrombus with a non-persistent CoNS bacteremia, 2/18 received rifampin because of increasing CRP levels during vancomycin therapy in combination with severe thrombocytopenia and a serious suspicion of an intravascular thrombus.

**Figure 2:**
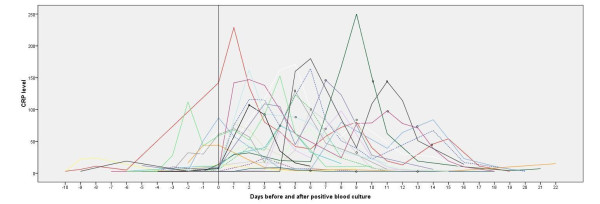
**CRP levels (mg/L) from 10 days before positive blood culture until maximum of 10 days after negative blood culture**. Each line indicates an individual patient; dotted lines represent infants with persistent CoNS sepsis without rifampin therapy. Start of rifampin therapy is indicated with circles.

Figure 2 shows the course of the CRP levels before and after the first CoNS positive blood culture, for both infants treated with and without rifampin (start of rifampin is marked, dotted lines represent infants without rifampin therapy).

**Figure 3:**
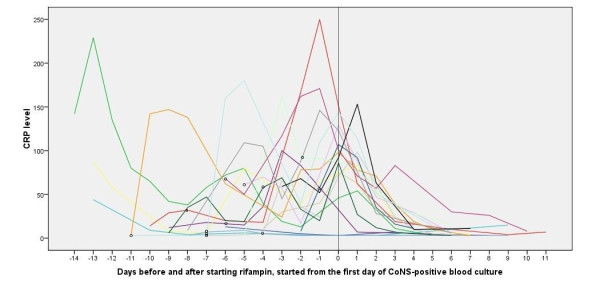
**CRP levels (mg/L) from the first day of CoNS-positive blood culture until 10^th ^day after starting rifampin treatment**. Each line indicates an individual patient, the time when indwelling catheters were removed is indicated with a circle.

CRP levels from the first day of a CoNS positive blood culture until the tenth day of rifampin therapy demonstrate a serious decline in CRP levels after starting rifampin therapy (figure 3). Indwelling catheters were removed before the first CoNS positive blood culture (6 times), on the day of the first CoNS positive blood culture (3 times) or after the first CoNS blood culture (6 times, although in all these infants maximum CRP was achieved after removal of the catheter). The most important decline in CRP occurred during the first 3 days after the start of rifampin. In these first 3 days the blood culture of most neonates became sterile, with a mean duration of 2.3 ± 1.6 days. Values of other infection- and pharmacokinetic parameters of the neonates treated with rifampin are listed in table 2.

**Table 2:** Infection and pharmacokinetic parameters of neonates treated with rifampin.

N = 18	Mean	SD	Min	Max
Age at start of bacteremia	5.4	2.7	1	11

Days of bacteremia before start of rifampin	8.0	3.6	2	14

Days of vancomycin therapy before start of rifampin	8.9	4.5	2	20

Maximum peak level of vancomycin (mg/L)	26.7	3.5	20.0	32.0

Minimum peak level of vancomycin (mg/L)	20.3	4.7	12.8	28.0

Maximum trough level of vancomycin (mg/L)	10.8	2.6	7.3	15.9

Minimum trough level of vancomycin (mg/L)	7.3	3.6	3.5	15.9

CRP at start of rifampin (mg/L)	77.5	44.8	3	146

Maximum CRP during bacteremia (mg/L)	120.3	64.4	9	250

Duration of rifampin therapy (hours)	147.2	52.5	52	264

Dose of rifampin (mg/kg/day)	10.4	3.0	6.0	17.0

Rapidity of sterilization of blood culture after start of rifampin (days)	2.3	1.6	0	6

Total duration of bacteremia (days)	10.3	3.7	4	15

**Figure 4:**
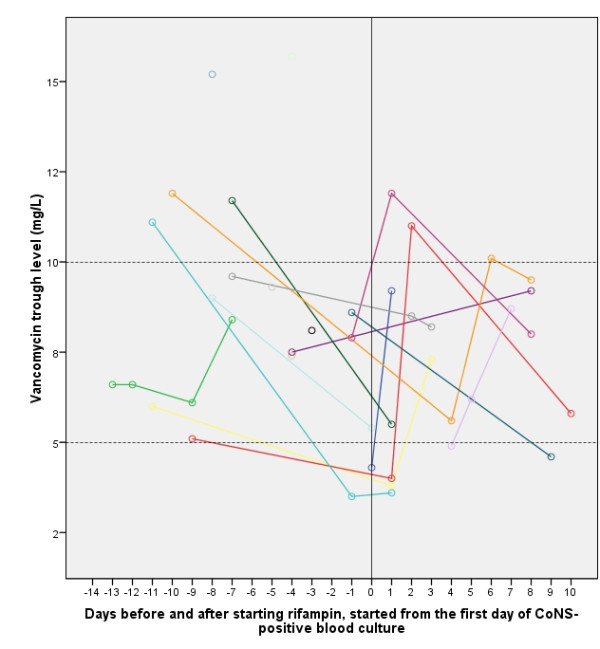
**Vancomycin trough levels (mg/L) of rifampin treated infants from the first day of CoNS-positive blood culture until 10^th ^day after starting rifampin treatment**. Each line indicates an individual patient.

Before the start of rifampin all neonates received vancomycin as monotherapy (n = 1) or in combination with ceftazidim (until definitive identification and antimicrobial susceptibility testing of gram-positive cocci in clusters) (n = 17). The duration of vancomycin therapy was 8.9 ± 4.5 days. Ten neonates had adequate initial vancomycin levels, in 8 infants vancomycin dosage had to be readjusted. Vancomycin trough levels between the first day of CoNS positive blood culture and the tenth day of rifampin treatment are presented in figure 4. In contrast to vancomycin levels, rifampin levels were never obtained.

The presence of an intravascular thrombus did not correlate with the total duration of CoNS bacteremia or with the rapidity of sterilization of blood cultures after the start of rifampin treatment.

## Discussion

Comparing our incidence of CoNS bacteremia (8%) with other studies is difficult, as the composition of study populations vary. Most studies report a lower incidence, probably due to higher birth weights and gestational ages in these populations [2-4]. One study reported an incidence of 19.9%, but neonates in this study had a lower gestational age [5]. Effectiveness of rifampin treatment in persistent staphylococcal bacteremia in neonates has been demonstrated in several case reports and pharmacokinetic studies, in which speed of sterilization of the blood culture was the main outcome [1,6-8]. Our data also show a substantial decline in CRP during the first days of rifampin treatment. To our knowledge, studies evaluating the treatment of CoNS bacteremia, focusing on the adequacy of monitoring and responding to vancomycin trough levels and the compliance with starting rifampin after 3 positive blood cultures with an interval of 48 hours, have not been reported earlier.

This retrospective study has several limitations. The most important one is the small size of the study population (18 patients) available for evaluation of rifampin treatment. As most neonates with a CoNS bacteremia respond well to vancomycin, rifampin is given only incidentally. Another limitation is the absence of an appropriate control group. As 4 patients with a persistent CoNS bacteremia did not receive rifampin, this group was too small for comparison purposes. Comparison of the occurrence of vancomycin levels below the therapeutic margin between persistent and non persistent bacteremia appeared difficult, as vancomycin levels were not regularly assessed in all neonates, especially in neonates with a non-persistent bacteremia.

Comparing the groups with and without persistent CoNS bacteremia, significant differences were seen for birth weight and the presence of hyperglycemia. Hyperglycemia is caused by relative insulin deficiency and resistance, due to high levels of circulating cytokines and inflammatory markers during sepsis [27]. Persistent bacteremia may increase the risk for developing co-morbidity such as hyperglycemia.

No clear statements can be made about the possible influence of adequate monitoring and the response to vancomycin trough levels on the risk of developing a persistent CoNS bacteremia. Because vancomycin levels were not consistently obtained in all neonates (especially in those without persistent CoNS-bacteremia), an accurate comparison of the occurrence of subtherapeutic vancomycin trough levels of infants with and without a persistent CoNS bacteremia was not possible.

## Conclusion

Our results suggest that the treatment strategy for persistent staphylococcal bacteremia with rifampin may be effective, but can be optimized by improving the monitoring of vancomycin trough levels and minimizing the delay in starting rifampin treatment. If, in spite of adequate vancomycin levels, CoNS bacteremia becomes persistent, rifampin therapy may be started after 6 days of bacteremia (3 positive blood cultures with a 48 hours interval after each).

## List of abbreviations

BPD: Bronchopulmonary dysplasia; IVH: Intraventricular hemorrhage; NEC: Necrotizing enterocolitis; NICU: Neonatal intensive care unit; PVL: Periventricular leukomalacia; PROM: Prolonged rupture of membranes; RDS: Respiratory distress syndrome; CONS: Coagulase negative staphylococci; CRP: C-reactive protein.

## Competing interests

The authors declare that they have no competing interests.

## Authors' contributions

NMvdL participated in the design of the study, collected the data, performed the statistical analysis and drafted the manuscript. SJS conceived of the study and helped to correct the manuscript. FJW conceived of the study, participated in its design and coordination and helped to draft the manuscript. All authors read and approved the final manuscript.

## Pre-publication history

The pre-publication history for this paper can be accessed here:

http://www.biomedcentral.com/1471-2431/10/84/prepub

## References

[B1] Tan TQ Mason EO Jr Ou CN Kaplan SL Use of intravenous rifampin in neonates with persistent staphylococcal bacteremia Antimicrob Agents Chemother 1993 37 2401 2406 8285624 10.1128/aac.37.11.2401 PMC192398

[B2] Orsi GB d'Ettorre G Panero A Chiarini F Vullo V Venditti M Hospital-acquired infection surveillance in a neonatal intensive care unit Am J Infect Control 2009 37 201 203 10.1016/j.ajic.2008.05.009 19059676

[B3] Wu JH Chen CY Tsao PN Hsieh WS Chou HC Neonatal sepsis: a 6-year analysis in a neonatal care unit in Taiwan Pediatr Neonatol 2009 50 88 95 10.1016/S1875-9572(09)60042-5 19579754

[B4] Auriti C Maccallini A Di LG Di C Ronchetti MP Orzalesi M Risk factors for nosocomial infections in a neonatal intensive-care unit J Hosp Infect 2003 53 25 30 10.1053/jhin.2002.1341 12495682

[B5] Lahra MM Beeby PJ Jeffery HE Intrauterine inflammation, neonatal sepsis, and chronic lung disease: a 13-year hospital cohort study Pediatrics 2009 123 1314 1319 10.1542/peds.2008-0656 19403497

[B6] Pullen J Stolk LM Degraeuwe PL van Tiel FH Neef C Zimmermann LJ Pharmacokinetics of intravenous rifampicin (rifampin) in neonates Ther Drug Monit 2006 28 654 661 10.1097/01.ftd.0000245382.79939.a4 17038881

[B7] Shama A Patole SK Whitehall JS Intravenous rifampicin in neonates with persistent staphylococcal bacteraemia Acta Paediatr 2002 91 670 673 10.1080/080352502760069098 12162600

[B8] Soraisham AS Al-Hindi MY Intravenous rifampicin for persistent staphylococcal bacteremia in premature infants Pediatr Int 2008 50 124 126 10.1111/j.1442-200X.2007.02519.x 18279222

[B9] Watanakunakorn C Guerriero JC Interaction between vancomycin and rifampin against Staphylococcus aureus Antimicrob Agents Chemother 1981 19 1089 1091 7271276 10.1128/aac.19.6.1089 PMC181616

[B10] Varaldo PE Debbia E Schito GC In vitro activity of teichomycin and vancomycin alone and in combination with rifampin Antimicrob Agents Chemother 1983 23 402 406 6221692 10.1128/aac.23.3.402 PMC184660

[B11] Bayer AS Morrison JO Disparity between timed-kill and checkerboard methods for determination of in vitro bactericidal interactions of vancomycin plus rifampin versus methicillin-susceptible and -resistant Staphylococcus aureus Antimicrob Agents Chemother 1984 26 220 223 6567464 10.1128/aac.26.2.220 PMC284124

[B12] Tuazon CU Lin MY Sheagren JN In vitro activity of rifampin alone and in combination with nafcillin and Vancomycin against pathogenic strains of Staphylococcus aureus Antimicrob Agents Chemother 1978 13 759 761 666300 10.1128/aac.13.5.759 PMC352328

[B13] Acar JF Goldstein FW Duval J Use of rifampin for the treatment of serious staphylococcal and gram-negative bacillary infections Rev Infect Dis 1983 5 Suppl 3 S502 S506 6635440 10.1093/clinids/5.supplement_3.s502

[B14] Zinner SH Lagast H Klastersky J Antistaphylococcal activity of rifampin with other antibiotics J Infect Dis 1981 144 365 371 6270215 10.1093/infdis/144.4.365

[B15] Wehrli W Rifampin: mechanisms of action and resistance Rev Infect Dis 1983 5 Suppl 3 S407 S411 6356275 10.1093/clinids/5.supplement_3.s407

[B16] Acocella G Pharmacokinetics and metabolism of rifampin in humans Rev Infect Dis 1983 5 Suppl 3 S428 S432 6356276 10.1093/clinids/5.supplement_3.s428

[B17] Mandell GL Vest TK Killing of intraleukocytic Staphylococcus aureus by rifampin: in-vitro and in-vivo studies J Infect Dis 1972 125 486 490 5023643 10.1093/infdis/125.5.486

[B18] Koup JR Williams-Warren J Weber A Smith AL Pharmacokinetics of rifampin in children. I. Multiple dose intravenous infusion Ther Drug Monit 1986 8 11 16 10.1097/00007691-198603000-00003 3961887

[B19] Giedion A Haefliger H Dangel P Acute pulmonary X-ray changes in hyaline membrane disease treated *with artificial ventilation and positive end-expiratory pressure (PEP)* Pediatr Radiol 1973 1 145 152 10.1007/BF00974058 4589887

[B20] Martin RJ Fanaroff AA Walsh MC Fanaroff and Martin's Neonatal-Perinatal Medicine: Diseases of the Fetus and Infant 2005 Philadelphia: Elsevier

[B21] Walsh MC Kliegman RM Necrotizing enterocolitis: treatment based on staging criteria Pediatr Clin North Am 1986 33 179 201 3081865 10.1016/S0031-3955(16)34975-6 PMC7131118

[B22] Sie LT van der Knaap MS van Wezel-Meijler G Taets van Amerongen AH Lafeber HN Valk J Early MR features of hypoxic-ischemic brain injury in neonates with periventricular densities on sonograms AJNR Am J Neuroradiol 2000 21 852 861 10815660 PMC7976754

[B23] Volpe JJ Neurology of the Newborn 2008 Philadelphia: Saunders

[B24] Papile LA Burstein J Burstein R Koffler H Incidence and evolution of subependymal and intraventricular hemorrhage: a study of infants with birth weights less than 1,500 gm J Pediatr 1978 92 529 534 10.1016/S0022-3476(78)80282-0 305471

[B25] Grootendorst DC de Jager DJ Brandenburg VM Boeschoten EW Krediet RT Dekker FW NECOSAD Study Group Excellent agreement between C-reactive protein measurement methods in end-stage renal disease patients--no additional power for mortality prediction with high-sensitivity CRP Nephrol Dial Transplant 2007 22 3277 3284 10.1093/ndt/gfm381 17623721

[B26] de Hoog M Schoemaker RC Mouton JW van den Anker JN Vancomycin population pharmacokinetics in neonates Clin Pharmacol Ther 2000 67 360 367 10.1067/mcp.2000.105353 10801244

[B27] Beardsall K Dunger D Insulin therapy in preterm newborns Early Hum Dev 2008 84 839 842 10.1016/j.earlhumdev.2008.09.013 18848411

